# Supplementary material for: The performance of restricted AIC for irregular histogram models
Source: PLoS One. 2024 May 1;19(5):e0289822. doi: 10.1371/journal.pone.0289822 (PMC11062542; doi:10.1371/journal.pone.0289822)
Supplement: S1 File — (PDF) [file pone.0289822.s001.pdf]

# Supporting information

For $X$ random variable with an unknown density $f$, the ordered statistics denote as $x_{(1)} \leq x_{(2)} \leq \cdots \leq x_{(n)}$. Constructing a histogram with unequal bins for given $(x_1, \ldots, x_n)$ by penalized likelihood means maximizing the function below

$$L(\hat{f}_{\mathcal{I}}, x_1, \ldots, x_n) - pen_n(\mathcal{I}) \tag{1}$$

where $pen_n(\mathcal{I})$ is a penalty term depending on the partition $\mathcal{I} = (I_1, \ldots, I_{|\mathcal{I}|})$. When the partitions $\mathcal{I} := \mathcal{I}_{\mathcal{D}} := (I_1, \ldots, I_D)$ of the interval $I := [x_{(1)}, x_{(n)}]$ consider, consisting of $D$ intervals of the form

$$I_j := \begin{cases} [t_0, t_1] & j = 1 \\ (t_{j-1}, t_j] & j = 2, \ldots, D \end{cases}$$

with the breakpoints $x_{(1)} =: t_0 < t_1 < \cdots < t_D := x_{(n)}$. In that point, a histogram is called irregular or unequal if all the intervals have not the same length.

Based on these, with $D =| \mathcal{I} |$, some proposed methods through parameterized $c$ and $\alpha$ constants,

$$pen_n^A(\mathcal{I}) = c \log \binom{n-1}{D-1} + \alpha(D-1) + \epsilon_{c,\alpha}^{(1)}(D), \tag{2}$$

$$pen_n^B(\mathcal{I}) = c \log \binom{n-1}{D-1} + \alpha(D-1) + \epsilon^{(2)}(D) \tag{3}$$

$$pen_n(\mathcal{I}) = c_1 \left( D - 1 + c_2 \left( \log \binom{n-1}{D-1} + k \log D \right) + 2\sqrt{c_2(D-1) \left( \log \binom{n-1}{D-1} + k \log D \right)} \right) \tag{4}$$

where 2 and 3 proposed by [1] while 4 proposed by [2] and

$$\epsilon_{c,\alpha}^{(1)}(D) = ck \log D + 2\sqrt{c\alpha(D-1) \left( \log \binom{n-1}{D-1} + k \log D \right)}, \tag{5}$$

$$\epsilon^{(2)}(D) = \log^{2.5} D.$$

If the notations consider with $\alpha = c_1, c = c_1 c_2$, Equation 4 reaches Equation 5.

According to these explanation, the methods compared in the simulations are:

- B represents penalized ML using penalty 2 with $c = 1, \alpha = 1$ while R represents penalized ML using penalty 3 with $c = 1$, $\alpha = 0.5$. Then CV represents leave-one-out cross-validation using 5 given by [3]. Also, maximization performed without restriction on the minimum bin width.
- Bc, Rc and CVc methods obtained in the same way with B, R, CV but here, it used an additional constraint defined as the minimum bin width is $(x_{(n)} - x_{(1)}) \log^{1.5}(n)/n$.
- AIC represents Akaike's Information Criteria [4] and its penalty is $pen_n^{AIC}(D) = (D - 1)$.
- BIC shows Bayesian Information Criteria [5] and its penalty is $pen_n^{BIC}(D) = 0.5 \log(n)(D - 1)$.
- TS which means taut string method in short, produced by [6]. In this method, the histogram is constructed by a spline with knots at the points at which it touches the lower or upper boundaries of the bins.

## References


1. Rozenholc Y, Mildenberger T, Gather U. Combining regular and irregular histograms by penalized likelihood. Computational Statistics & Data Analysis. 2010;54(12):3313–3323.
2. Massart P. Lecture notes in Mathematics Vol. 1896: Concentration Inequalities and Model Selection; 2007.
3. Celisse A, Robin S. Nonparametric density estimation by exact leave–p-out cross–validation. Computational Statistics & Data Analysis. 2008;52(5):2350–2368.
4. Akaike H. Information theory and an extension of the maximum likelihood principle. In: Parzen E, Tanabe K, Kitagawa G, editors. Selected papers of Hirotugu Akaike. New York: Springer; 1998. p. 199–213.
5. Schwarz G. Estimating the dimension of a model. The annals of statistics. 1978;6(2):461–464.
6. Davies PL, Kovac A, et al. Densities, spectral densities and modality. The Annals of Statistics. 2004;32(3):1093–1136.
