# Supplementary material for: The performance of restricted AIC for irregular histogram models
Source: PLoS One. 2024 May 1;19(5):e0289822. doi: 10.1371/journal.pone.0289822 (PMC11062542; doi:10.1371/journal.pone.0289822)
Supplement: Attachment — Submitted filename: response to reviewers.docx [file pone.0289822.s002.docx]

To Editor:

1- Even we used the PLOS ONE’s Latex template (<https://journals.plos.org/plosone/s/latex>), we revised the style of manuscript based on your directions (affiliations, main body, headlines etc.).
2- Title page that includes all authors and affiliations merged with main text.
3- All the dataset and codes now available in the following link (DOI: 10.5281/zenodo.8059567 ). Also this information is added to the cover letter.

<https://zenodo.org/record/8059567>

4- Financial disclosure information is updated with the statement of “The funders had no role in study design, data collection and analysis, decision to publish, or preparation of the manuscript.”
5- The reference list is checked and as far as we know there is no retracted paper that has been cited in this paper.

To Reviewers:

First of all, we want to thank the reviewers for substantially improving the manuscript! Please find each of the comments below with our response.

Reviewer 1:

1- Based on the suggestion of the reviewer, the novelty, research gap and the main contributions of the paper are rewritten in the Introduction section.
2- We searched the literature again and haven’t found anything more recent about the related field.
3- The weak performances of proposed AICr are implied both in Monte Carlo and Conclusion sections.
4- Again, unfortunately we searched the literature again and haven’t found anything more recent about the related field.

Reviewer 2:

Grammatical errors are corrected in the text.
